# Serum Osteoprotegerin Level Is Not a Localizing Biomarker of Atherosclerosis Affected by Kidney Function

**DOI:** 10.3390/diagnostics16050786

**Published:** 2026-03-06

**Authors:** Anna Maria Bednarek, Aleksander Jerzy Owczarek, Dominika Dziadosz, Magdalena Olszanecka-Glinianowicz, Jerzy Tadeusz Chudek

**Affiliations:** 1 Department of Cardiac Surgery, Upper Silesian Heart Center, 40-635 Katowice, Poland; 2 Health Promotion and Obesity Management Unit, Department of Pathophysiology, Faculty of Medical Sciences in Katowice, Medical University of Silesia in Katowice, 40-751 Katowice, Poland; aowczarek@sum.edu.pl (A.J.O.); magolsza@gmail.com (M.O.-G.); 3 First Department of Cardiology, Faculty of Medical Sciences in Katowice, Medical University of Silesia in Katowice, 40-635 Katowice, Poland; dominika.dziadosz@gmail.com; 4 Department of Internal Medicine and Oncological Chemotherapy, Faculty of Medical Sciences in Katowice, Medical University of Silesia in Katowice, 40-029 Katowice, Poland; chj@poczta.fm; 5 Angiology Outpatient Clinic “Combi-Med”, 42-218 Czestochowa, Poland

**Keywords:** osteoprotegerin, atherosclerosis, abdominal aorta aneurysm

## Abstract

**Introduction**: Osteoprotegerin (OPG) is recognized as an emerging biomarker for atherosclerosis. We hypothesized that atherosclerotic lesions localized across multiple vascular beds would result in greater elevations in OPG levels in the blood. Therefore, our study aimed to assess serum OPG levels and their confounding factors in patients with hemodynamically significant multivessel atherosclerosis in varying locations. **Subjects and Methods**: A case–control study included 222 selected outpatients aged 50 years or older (46.4% women) with atherosclerosis confirmed by imaging (Doppler ultrasound and CT angiography) treated at a single angiology clinic. Data concerning age, smoking status, comorbidity (hypertension, diabetes mellitus, history of stroke, myocardial infarction, coronary revascularization procedures), medication, lipid profile, serum creatinine, and homocysteine levels were retrieved from medical records. Additionally, serum OPG levels were measured. Patients were divided according to serum OPG levels into terciles and the number of involved vascular beds [carotid artery disease, coronary heart disease (CHD), lower-extremity peripheral artery disease (PAD), abdominal aorta aneurysm (AAA)]. **Results**: The distribution of carotid artery disease, CHD, PAD, and AAA did not differ across the OPG terciles. Additionally, we did not observe differences in OPG levels between specific and multiple locations of atherosclerotic lesions. Subjects with the highest OPG levels were the oldest (75.0 ± 8.4 vs. 69.8 ± 7.1 years in the lowest tercile; *p* < 0.001) and were characterized by the worst kidney function (eGFR 60.8 ± 16.8 vs. 74.1 ± 13.5 mL/min/1.73 m^2^; *p* < 0.001). **Conclusions**: The serum OPG level did not reveal the specific location of atherosclerosis. Impaired renal function appears to be the primary determinant of serum OPG levels and a key confounder, complicating the interpretation of serum OPG as a biomarker of atherosclerosis.

## 1. Introduction

Osteoprotegerin (OPG), a soluble ‘decoy receptor’ for the receptor activator of nuclear factor kappa B ligand (RANKL) and TNF-related apoptosis-inducing ligand (TRAIL), is considered an emerging atherosclerosis biomarker (BM) [1,2,3]. Higher serum/plasma OPG levels were reported in patients with coronary heart disease (CHD) [4,5], heart failure after past myocardial infarction, aortic aneurysms [6], peripheral artery disease (PAD) [7], past stroke [8], and even valvular heart diseases [9]. Additionally, serum OPG levels are proportional to increasing carotid intima-media thickness (CIMT), arterial stiffness, aortic pulse wave velocity (aPWV), and severity of atherosclerosis [10,11].

The circulating OPG reflects its production by bone marrow stromal cells, lymphocytes B, dendritic cells, endothelial cells (ECs), and vascular smooth muscle cells (VSMCs). During the early stage of atherosclerosis, OPG is extensively produced by ECs, revealing endothelial dysfunction [12]. Constitutively produced OPG by VSMCs is upregulated by proinflammatory cytokines. Although the exact mechanism is uncertain, it is expected that the ability of VSMCs to produce OPG is maintained during their transition into myofibroblasts and ultimately calcifying vascular cells (CVCs). In vivo, TGF-beta1 synergistically enhanced fibroblast responses to elastin degradation products, leading to increased expression of bone-regulating proteins, including core-binding factor alpha1, osteocalcin, alkaline phosphatase, and OPG [13]. The production of OPG seems highest in moderately calcified plaques, as shown by Higgins et al. [14]. Moreover, they revealed that with progressive atherosclerotic plaque calcification, tissue levels of OPG decline, being lowest in calcified human carotid plaques, as supported by an inverse association between OPG in calcified human carotid plaques and calcification severity measures, including calcium hydroxyapatite content and the Agatstone score (r = −0.432 and −0.579) [15]. In this study, tissue and serum OPG levels were found to be strongly associated (r = 0.820). These make OPG a vital biomarker of non-calcified, unstable, and vulnerable plaques, which are of high significance for cardiovascular and cerebrovascular events. Moreover, increased OPG concentrations were shown to predict the occurrence of cardiovascular disease [15]. Furthermore, more than twice as high serum OPG levels were reported in diabetic patients with unstable rather than stable ICA stenosis [16].

Atherosclerotic processes across all vascular beds share many similarities and frequently co-occur, resulting in multivessel atherosclerosis. The differences are primarily attributable to the histological structure of the arterial wall, blood flow, and increased endothelial shear stress. We hypothesized that the involvement of multiple vascular beds would result in a greater increase in OPG concentration in the circulation. Therefore, our study aimed to assess serum OPG levels and their confounding factors in patients with hemodynamically significant multivessel atherosclerosis in varying locations.

## 2. Materials and Methods

This single-center case–control study included 222 selected Caucasian patients (age range: 50–92 years) with atherosclerosis confirmed by imaging techniques (Doppler ultrasound and CT angiography), recruited from the outpatient department of the angiology clinic from October 2018 to December 2020.

The study protocol, which included the use of frozen samples left over from routine diagnostic procedures for additional testing, was approved by the Bioethical Committee of the Medical University of Silesia (KNW/022/KB1/90/18).

Patients with moderate-to-severe and severe chronic kidney disease (CKD) with an estimated glomerular filtration rate < 45 mL/min/1.73 m^2^, liver cirrhosis, gastrointestinal diseases with malabsorption, dementia precluding obtaining informed consent, and overt infection were excluded.

Data on performed imaging that confirmed diagnoses of atherosclerosis (carotid artery disease, lower-extremity peripheral artery disease—PAD, abdominal aortic aneurysm—AAA) and standard laboratory tests (lipid profile, homocysteine, creatinine) were retrieved from medical records. AAA was defined as local dilation of the aorta diameter > 30 mm in CT angiography. PAD was defined as at least 50% stenosis of the iliac, femoral, or popliteal arteries in CT angiography. The diagnosis of carotid artery disease was based on Doppler ultrasound demonstrating a peak systolic velocity (PSV) > 1.25 m/s, in accordance with consensus criteria [16]. Coronary heart disease (CHD) was established based on past acute myocardial infarcts and revascularization procedures.

The only study procedure beyond routine diagnostics was the assessment of serum OPG levels obtained from blood drawn in the morning in a fasting state. Serum samples were stored frozen at −40 °C until being transported to the Laboratory of the Department of Pathophysiology.

Additional assessments were performed on shortly stored frozen samples (2020–2021) in the Laboratory of the Department of Pathophysiology. Serum concentrations of OPG were quantified in duplicates using available ELISA kits from BioVendor (Brno, Czech Republic), with intra- and inter-assay coefficients of variation of <4.9% and <9%, respectively.

### 2.1. Data Analysis

Diagnosis of past stroke was based on discharge cards from neurology departments. Other established comorbidities (hypertension and diabetes mellitus) and medication were based on medical records.

Patients were stratified into terciles based on serum OPG concentration (lower: 9.12 pmol/L; upper: 12.2 pmol/L). We aim to determine whether a specific location of atherosclerosis affects serum OPG levels and to detect confounding factors.

### 2.2. Statistical Analysis

Statistical analysis was performed using STATISTICA 13.0 PL (Tibco Software Inc., Palo Albo, CA, USA) and Stata SE 12.0 (StataCorp LP, College Station, TX, USA). A *p*-value of less than 0.05 determined statistical significance. All tests were two-tailed. Imputations were not done for missing data. Nominal and ordinal data were expressed as numbers and percentages, while interval data were expressed as mean values ± standard deviation in the case of a normal distribution or as the medians (lower quartile; upper quartile) in the case of data with skewed or non-normal distribution. The distribution of variables was evaluated by the Shapiro–Wilk test and the quantile–quantile (Q-Q) plot. The homogeneity of variances was assessed by the Fisher–Snedecor test. A one-way ANOVA with Dunnett’s test as the post hoc test was used to compare data across OPG terciles and between atherosclerosis locations. Between the older and younger groups, the Student t-test was used for normally distributed data. For skewed data, a logarithmic transformation was applied. Data in nominal and ordinal scales were compared with the χ^2^ test and log-linear analysis. The Spearman rank correlation (and partial correlation) was used to measure associations between covariates.

## 3. Results

### 3.1. Study Group Characteristics

The most frequent localization of atherosclerosis in the study group was lower-extremity PAD—57.2% (N = 127), followed by carotid artery disease—36.9% (N = 82), AAA—26.6% (N = 59), and CHD—23.4% (N = 52), reflecting the typical profile for angiology outpatient clinics. Atherosclerosis was diagnosed at more than one site (typically 2 or 3) in 38.7% (N = 86) of patients.

Three of ten (N = 67) were treated for diabetes, and 65.3% (N = 145) for arterial hypertension. A decreased eGFR (<60 mL/min/1.73 m^2^), characteristic of chronic kidney disease, was observed in 26.1% (N = 58). Statins and ezetimibe were prescribed in 89.2% (N = 198) and 11.7% (N = 26) of cases, respectively.

The serum OPG level was 10.7 (lower quartile—Q1; upper quartile—Q3: 8.3; 13.5) pmol/L, without a significant difference between men and women: 10.8 pmol/L (Q1; Q3: 8.1; 13.6) and 10.6 pmol/L (Q1; Q3: 8.8; 13.4), *p* = 0.91.

### 3.2. Distribution of Atherosclerosis Location According to Serum OPG Terciles

The distribution of carotid artery disease, CHD, lower-extremity PAD, and AAA did not differ across the OPG terciles (Table 1). Subjects with the highest serum OPG levels were the oldest, characterized by the poorest kidney function, the highest rate of decreased eGFR (<60 mL/min/1.73 m^2^), and increased homocysteine levels.

### 3.3. Serum OPG in Relation to Atherosclerosis Localization

**Figure 1:**
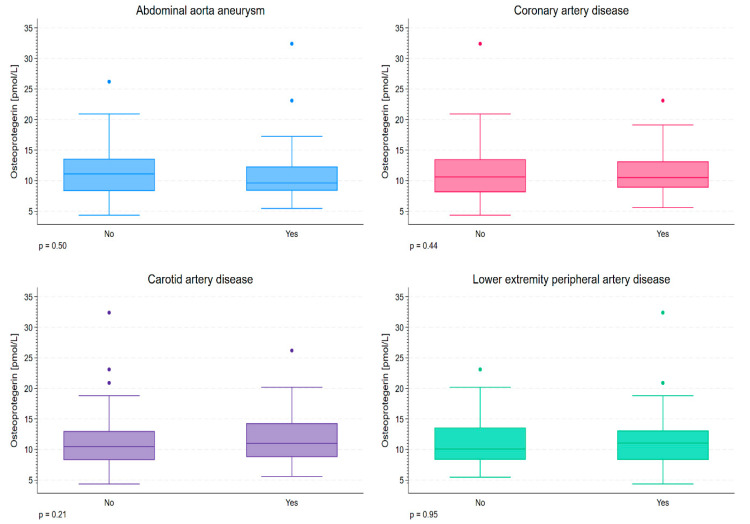
Serum osteoprotegerin levels stratified according to the occurrence of abdominal aorta aneurysm, coronary heart disease (**upper panel**), carotid artery disease, and lower-extremity peripheral artery disease (**lower panel**). The differences were statistically not significant. *p*-values are shown below the panels.

We did not detect differences in serum OPG levels associated with specific locations of hemodynamically significant atherosclerotic lesions (Figure 1). In addition, similar levels were observed in patients with atherosclerotic involvement of a single vascular bed and two or more vascular beds: 10.6 pmol/L (Q1; Q3: 8.1; 13.2) and 10.8 pmol/L (Q1; Q3: 9.0; 13.7), respectively, *p* = 0.24.

### 3.4. Factors Affecting Serum OPG Levels

Age (σ = 0.34; *p* < 0.001) and eGFR (mL/min/1.73 m^2^) (σ = −0.35; *p* < 0.001) were identified as significant, albeit weak, correlates of serum OPG levels. The correlation with age, after adjustment for eGFR and statin use, lost strength but remained significant (σ = 0.23; *p* < 0.01).

**Figure 2:**
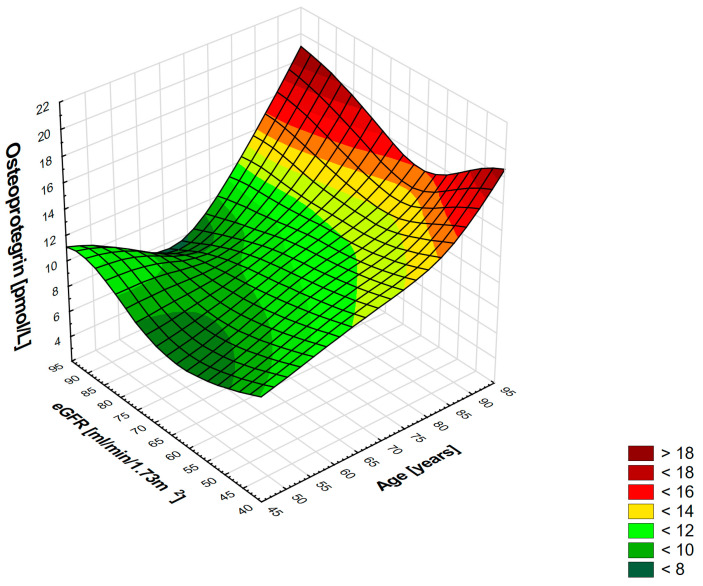
Interrelation between estimated glomerular filtration rate (eGFR), age, and osteoprotegerin (OPG) levels. The three-dimensional surface plot illustrates the association between circulating OPG levels, age, and eGFR. The surface is color-coded to represent different OPG concentration ranges, transitioning from dark green (lowest levels) to lighter green and yellow to red (highest levels). Overall, the figure demonstrates that OPG concentrations tend to increase with advancing age and decreasing eGFR. The lowest OPG levels are observed in younger individuals with higher eGFR values, as indicated by the green regions on the surface. As age increases and renal function declines, OPG concentrations progressively rise, as indicated by a shift toward the yellow and red regions. The highest OPG values are seen in the oldest individuals with markedly reduced eGFR, suggesting a combined effect of aging and renal impairment on OPG levels. The color legend alongside the plot further categorizes OPG concentrations into discrete ranges, reinforcing the gradient pattern observed across age and kidney function. The plot also shows a group of older patients with elevated OPG levels and inappropriately high-for-age eGFR values, reflecting low muscle mass and thereby distorting GFR estimates with creatinine-based equations.

The three-dimensional surface plot illustrates the association between circulating OPG levels, age, and eGFR (Figure 2). Osteoprotegerin concentrations (vertical axis) demonstrate a nonlinear relationship with both age (*x*-axis) and eGFR (*y*-axis). The curved surface indicates that OPG levels increase more steeply at lower eGFR values and at older age.

The highest serum OPG levels were observed in the oldest group (≥75 years old), with almost three times higher rates of eGFR < 60 mL/min/1.73 m^2^—Table 2. This subgroup of patients had a higher prevalence of AAA and CHD, as well as higher serum homocysteine levels.

There was a weak correlation between serum OPG and homocysteine levels (σ = 0.21; *p* < 0.01) that lost its significance after adjustment for eGFR (mL/min/1.73 m^2^) (σ = 0.10; *p* = 0.2).

## 4. Discussion

We showed that subjects with various locations of atherosclerosis present with similarly increased serum OPG levels, and that the clinical involvement of more than one localization (vascular bed) by atherosclerosis does not result in higher levels. Therefore, OPG cannot be considered a measure of the extent of hemodynamically significant atherosclerosis (multivessel atherosclerosis).

In addition, we confirmed previous observations of an age-related increase in serum OPG levels [1,2,3], which is partially related to declining eGFR. The negative association between circulating OPG levels and eGFR was demonstrated in the Chronic Renal Insufficiency Cohort (CRIC) [17] and in patients with stages 2 to 5 of chronic kidney disease not requiring dialysis treatment [18]. There is limited data on the kinetics and clearance of OPG in humans and animals. Biodegradation of OPG in the bones is mediated primarily by proteolytic enzymes such as cathepsin K and by cellular uptake by osteoclasts [19], while circulating OPG can be removed by the liver and kidneys, because in both liver cirrhosis [20] and chronic kidney disease [18], OPG levels are increased. The role of the kidney in the elimination of circulating OPG is supported by the correlation between OPG and homocysteine observed in our study, consistent with simultaneous changes in eGFR decline.

Numerous previous studies have analyzed the association between the specific localization of atherosclerosis and OPG levels in the circulation but have not examined differences attributable to different vascular beds and multiple localizations, nor have they analyzed kidney function.

In patients with type 2 diabetes, aged 40 years or above, plasma OPG levels were shown to increase gradually with the severity of lower-extremity arterial stenosis assessed by Doppler sonography [21]. Unfortunately, kidney function was not reported in this study. Therefore, it remains unknown whether the differences could not be explained by eGFR decline in individuals with more severe arterial stenosis. Another study analyzed the prognostic significance of increased serum OPG levels in patients with type 1 diabetes during a 12-year follow-up [22]. This study showed an association of serum OPG levels with the development of foot ulcers, vascular surgery/amputations, and other chronic complications of diabetes, among them nephropathy. After multiple adjustments, including age and eGFR [23], only the association with the development of foot ulcers, which is more closely related to diabetic microangiopathy, remained significant. The serum OPG level was significantly associated with both the presence and severity of PAD in patients with T2D [23]. In Dakhel’s study [24], the authors looked for multiple markers of atherosclerosis in patients with aortic aneurysm. Osteoprotegerin was included among the 11 examined, but none demonstrated high diagnostic utility. Therefore, the significance of OPG as a specific biomarker of macroangiopathy/atherosclerosis severity is questionable.

Similarly, researchers investigating the association between serum OPG levels and the severity of carotid artery stenosis have shown higher levels in symptomatic than asymptomatic patients [18] and in those with unstable rather than stable atherosclerotic plaques [25]. Notwithstanding, higher serum OPG levels were shown in patients with calcified (more stable) rather than non-calcified carotid plaques [26]. The analysis of these discrepancies is complicated by the use of different methods for assessing OPG levels [27] and by the lack of adjustment for eGFR as a confounding factor.

The quantification of atherosclerotic lesions is most commonly performed for coronary arteries using the coronary artery calcium (CAC) score (calcium Agatston score) [28]. Data on the relationship between serum OPG concentration and CAC score are inconsistent. Some but not all [29,30] studies showed a positive correlation between serum OPG level and CAC score in patients with moderate–severe cardiovascular risk (r = 0.694) [31], rheumatoid arthritis (r = 0.27) [32], and chronic kidney disease (r = 0.377) [33]. The lack of adjustment for eGFR, with the exception of KNOW-CKD [33], and the lack of quantification of atherosclerotic lesions outside coronary arteries in these studies, make it difficult to draw an unambiguous conclusion.

It has been shown that circulating OPG is an independent long-term predictor of all-cause mortality and cardiovascular events in patients with carotid artery disease. Statin treatment was associated with lower OPG levels in patients without diabetes [34]. Increased OPG was associated with 15% higher CAC values after adjustment for major covariates [35]. Still, these findings preclude a strong relationship between atherosclerosis severity and serum OPG levels within a single vascular bed.

Our supposition that serum OPG levels may serve as a global biomarker of atherosclerosis (present across all vascular beds) is supported by their association with age. Kudlacek et al. [36] observed a sharp increase in serum OPG levels in females after the age of 60 years and in males after the age of 70 years. These findings reflect the prevalence of atherosclerosis in the population and support the potential use of serum OPG levels as a biomarker for identifying patients with cardiovascular disease. Recently, serum OPG levels have been proposed as biomarkers of geriatric frailty syndrome and age-associated organ damage [37]. These may explain the nonlinear association between circulating OPG and eGFR in the oldest subgroup in our study. Low muscle mass in frail adults causes overestimation of GFR [38].

Our cross-sectional study has limitations in assessing vascular bed involvement by atherosclerosis, specifically the lack of quantification of atherosclerotic extent, plaque thickness, and their calcification. However, multislice CT, as the most accurate method for quantifying atherosclerotic lesions, still has limited use outside the coronary arteries. In addition, we defined CHD, omitting patients with stable angina without prior coronary interventions and myocardial infarction episodes, as patients without confirmation of coronary arteriosclerosis in imaging. These can be sources of bias that led to an underestimation of coronary atherosclerosis in our study. Furthermore, the cross-sectional nature of our study precludes the causal and prognostic interpretation of OPG levels. However, our findings, along with the identified limitations of OPG assessment related to kidney function, should be considered when designing new projects.

## 5. Conclusions

This paper discusses the role of serum OPG levels as a potential localizing biomarker for atherosclerosis. It indicates that the serum OPG level did not reveal the specific location of atherosclerosis and did not increase further in multivessel atherosclerosis. In addition, the interpretation of serum OPG levels is confounded by variability in kidney function, even in patients with mild-to-moderate chronic kidney disease. Impaired renal function appears to be a key confounder of serum OPG levels when used as a biomarker of atherosclerosis.

## Figures and Tables

**Table 1:** Comparison of subgroups stratified based on osteoprotegerin concentration terciles (lower 9.12 pmol/L, upper 12.2 pmol/L).

	Lower Tercile	Middle Tercile	High Tercile	*p*
	*n* = 74	*n* = 72	*n* = 72	
Osteoprotegerin, pmol/L	7.8 (6.7; 8.4)	10.8 (9.8; 11.4)	14.5 (13.5; 16.1)	–
Females, *n* (%)	35 (47.3)	33 (45.8)	32 (44.4)	0.95
Age, years	69.8 ± 7.1	71.8 ± 6.9	76.0 ± 8.4 ^#^	<0.001
≥75 years, *n* (%)	14 (18.9)	25 (34.7) *	38 (52.8) ^#^	<0.001
Smokers, *n* (%)	40 (58.8)	26 (40.0)	28 (43.1)	0.06
Arterial hypertension, *n* (%)	50 (68.5)	49 (70.0)	46 (66.7)	0.91
Diabetes mellitus, *n* (%)	23 (31.1)	21 (29.6)	23 (33.3)	0.89
Past stroke, *n* (%)	8 (10.8)	10 (13.9)	9 (13.0)	0.84
Abdominal aorta aneurysm, *n* (%)	23 (31.1)	21 (29.2)	15 (20.8)	0.34
Carotid artery disease, *n* (%)	24 (32.4)	29 (40.3)	29 (40.3)	0.53
Lower-extremity peripheral artery disease, *n* (%)	43 (58.1)	42 (59.2)	42 (60.0)	0.97
Coronary heart disease, *n* (%)	15 (20.3)	18 (25.4)	19 (26.8)	0.63
Number of vascular beds involved, *n*	1.4 ± 0.6	1.5 ± 0.7	1.5 ± 0.6	0.60
≥2 vascular beds locations, *n* (%)	25 (33.8)	31 (43.1)	30 (41.7)	0.46
Total cholesterol, mg/dL	166.6 ± 41.7	166.3 ± 46.1	161.3 ± 42.8	0.72
LDL-cholesterol, mg/dL	81.2 ± 33.9	78.7 ± 36.4	78.3 ± 35.3	0.86
HDL-cholesterol, mg/dL	63.5 ± 16.1	61.4 ± 14.9	61.4 ± 13.7	0.64
Triglycerides, mg/dL	104.5 (77.0; 151.0)	106.0 (89.0; 143.0)	106.0 (89.0; 143.0)	0.70
Homocysteine, µmol/L	15.9 ± 5.6	16.7 ± 5.3	19.3 ± 7.2 **	<0.01
eGFR_CKD-EPI_, mL/min/1.73 m^2^	74.1 ± 13.5	70.6 ± 15.4	60.8 ± 16.8 ^#^	<0.001
<60 mL/min/1.73 m^2^, *n* (%)	11 (17.7)	14 (23.3)	33 (49.2) ^#^	<0.001
Lipid-lowering therapy				
Statin, *n* (%)	69 (93.2)	67 (93.1)	62 (86.1)	0.24
Ezetymib, *n* (%)	11 (14.9)	8 (11.1)	7 (9.7)	0.61

Data presented as mean value ± standard deviation or median (lower quartile; upper quartile). Comparison to the lowest tercile: * *p* < 0.05, ** *p* < 0.01, ^#^ *p* < 0.001.

**Table 2:** Comparison of age subgroups. The age of 75 years was the value of the third tercile.

	Age < 75 Years	Age ≥ 75 Years	*p*
***n*** **(%)**	**142 (64.0)**	**80 (36.0)**	–
Osteoprotegerin, pmol/L	9.7 (7.8; 11.8)	12.2 (9.8; 14.5)	<0.001
Age, years	68.0 ± 5.7	80.7 ± 7.0	–
Females, *n* (%)	70 (49.3)	33 (41.3)	0.28
Smokers, *n* (%)	71 (54.6)	26 (36.1)	<0.05
Arterial hypertension, *n* (%)	92 (66.7)	57 (73.1)	0.33
Diabetes mellitus, *n* (%)	38 (27.1)	31 (39.7)	0.06
Past stroke, *n* (%)	16 (11.3)	12 (15.6)	0.37
Abdominal aorta aneurysm, *n* (%)	29 (20.4)	30 (37.5)	<0.01
Carotid artery disease, *n* (%)	52 (36.6)	30 (38.0)	0.84
Lower-extremity peripheral artery disease, *n* (%)	89 (63.1)	41 (53.2)	0.16
Coronary heart disease, *n* (%)	26 (18.4)	27 (34.2)	<0.01
Number of vascular beds involved, *n*	1.4 ± 0.6	1.6 ± 0.7	<0.05
≥2 vascular beds locations, *n* (%)	47 (33.1)	39 (48.8)	<0.05
Total cholesterol, mg/dL	167.8 ± 44.8	160.5 ± 40.0	0.23
LDL-cholesterol, mg/dL	82.8 ± 37.3	74.2 ± 29.0	0.06
HDL-cholesterol, mg/dL	62.3 ± 15.4	61.4 ± 13.7	0.65
Triglycerides, mg/dL	107.5 (81.0; 149.0)	105.0 (79.0; 150.0)	0.91
Homocysteine, µmol/L	16.2 ± 15.6	19.4 ± 18.3	<0.001
eGFR_CKD-EPI_, mL/min/1.73 m^2^	72.7 ± 15.8	60.8 ± 14.4	<0.001
<60 mL/min/1.73 m^2^, *n* (%)	21 (17.6)	39 (52.7)	<0.001
Lipid-lowering therapy			
Statin, *n* (%)	133 (93.7)	68 (85.0)	<0.05
Ezetymib, *n* (%)	24 (16.9)	3 (3.8)	<0.01

Data are presented as mean value ± standard deviation or median (lower quartile; upper quartile).

## Data Availability

The data are available from the corresponding author upon request.
