# Correction: Bednarek et al. Serum Osteoprotegerin Level Is Not a Localizing Biomarker of Atherosclerosis Affected by Kidney Function. *Diagnostics* 2026, *16*, 786

**DOI:** 10.3390/diagnostics16071040

**Published:** 2026-03-30

**Authors:** Anna Maria Bednarek, Aleksander Jerzy Owczarek, Dominika Dziadosz, Magdalena Olszanecka-Glinianowicz, Jerzy Tadeusz Chudek

**Affiliations:** 1 Department of Cardiac Surgery, Upper Silesian Heart Center, 40-635 Katowice, Poland; 2 Health Promotion and Obesity Management Unit, Department of Pathophysiology, Faculty of Medical Sciences in Katowice, Medical University of Silesia in Katowice, 40-751 Katowice, Poland; aowczarek@sum.edu.pl (A.J.O.); magolsza@gmail.com (M.O.-G.); 3 First Department of Cardiology, Faculty of Medical Sciences in Katowice, Medical University of Silesia in Katowice, 40-635 Katowice, Poland; dominika.dziadosz@gmail.com; 4 Department of Internal Medicine and Oncological Chemotherapy, Faculty of Medical Sciences in Katowice, Medical University of Silesia in Katowice, 40-029 Katowice, Poland; chj@poczta.fm; 5 Angiology Outpatient Clinic “Combi-Med”, 42-218 Czestochowa, Poland

In the original publication [1], the informed consent statement was marked as not applicable. After confirmation with the authors, the correct informed consent statement should be: Informed consent was obtained from all subjects involved in the study. 

The authors state that the scientific conclusions are unaffected. This correction was approved by the Academic Editor, and the original publication has also been updated.
